# PM_2.5_ Air Pollution and Cardiovascular Disease-Associated Disability among Middle-Aged and Older Adults

**DOI:** 10.5334/gh.1118

**Published:** 2022-06-16

**Authors:** Yanan Luo, Tao Xue, Yihao Zhao, Tong Zhu, Xiaoying Zheng

**Affiliations:** 1 Department of Global Health, School of Public Health, Peking University, Beijing, China; 2 Institute of Reproductive and Child Health, Peking University/Key Laboratory of Reproductive Health, National Health Commission of the People’s Republic of China, Beijing, China; 3 Department of Epidemiology and Biostatistics, School of Public Health, Peking University, Beijing, China; 4 Chinese Academy of Medical Sciences/Peking Union Medical College, School of Population Medicine and Public Health, China; 5 BIC-ESAT and SKL-ESPC, College of Environmental Science and Engineering, Peking University, Beijing, China

**Keywords:** ambient particles, cardiovascular diseases, disability, aged and older adults

## Abstract

**Background:**

Increasing evidence regards the role of ambient particles on morbidity and mortality caused by cardiovascular diseases (CVDs). However, there was no evidence about the association between ambient particles and CVD-associated disability. This study used large national representative data to investigate the relationship between long-term exposure to an aerodynamic diameter less than or equal to 2.5 µm (PM_2.5_) and CVD-associated disability among Chinese adults aged 45 years old and above and estimated the burden of CVD-associated disability attributed to PM_2.5_.

**Methods:**

Using data from the Second National Sample Survey on Disability, this study used a combination of self-reports or family members’ reports and on-site medical diagnosis by experienced specialists to ascertain CVD-associated disability in 852,742 adults aged 45 years old and above. Logistic regression models and spline regression models were used to examine the association between PM_2.5_ long-term exposure and CVD-associated disability, and the population attributable risk was calculated to assess the burden of CVD-associated disability contributed to PM_2.5_.

**Results:**

Every increase of 10 μg/m^3^ in PM_2.5_ was associated with an 8% (OR = 1.08, 95% CI: 1.05, 1.10) increase the odds of CVD-associated disability. Stratified analyses by demographic factors suggested that this association was robust. There were 1.05 (0.74,1.35) million -3.53 (3.29,3.75) million CVD-associated disabilities attributed to high PM_2.5_ concentration exposure (≥35 µg/m^3^) among middle-aged and older adults in 2006. A reduction in PM_2.5_ concentrations to 35 µg/m^3^ corresponded to a decrease of 13.59% (9.55%, 17.46%)–23.98% (17.17%, 30.25%) in CVD-associated disability by age group, respectively, and this magnitude increased in areas with a high prevalence of CVD-related disability.

**Conclusions:**

This study suggests that reducing PM_2.5_ concentrations may contribute to preventing CVD-associated disability and decreasing air pollution-related medical expenditures and rehabilitation fees.

## Introduction

Cardiovascular disease (CVD) is a major cause of disabilities later in life [1]. In 2017, CVD resulted in more than 360 million disability-adjusted life years (DALY) lost around the world [2]. As a permanently irreversible unhealthy condition, CVD-associated disability was also found to be a common health issue in both developed and developing countries [3]. In China, CVD poses substantial socioeconomic burdens on the economy and society [4]. Although the government has implemented several prevention and intervention strategies [4], the CVD prevalence has continued to rise and doubled since 1990. In 2016, the CVD prevalence reached nearly 94 million and led to the loss of over 78 million DALY [4].

There is increasing evidence regarding an association between ambient air pollution and CVD. As one of the common air pollutants, fine particulate matter (particulate matter of aerodynamic diameter ≤2.5 μm, PM_2.5_) has been considered the predominant air pollutant with significant effects on the circulatory system, such as an increase in the onset risk of stroke, myocardial infarction, heart failure, and cardiac arrest [5,6,7]. Additionally, exposure to PM_2.5_ was proven to be strongly associated with the increasing mortality of CVD [8]. In China, although the link between fine particulate matter and the risk of CVD has been suggested by previous studies [9,10,11], no study has further investigated the effects of PM_2.5_ on CVD-associated disability.

Apart from understanding the relationship between CVD and PM_2.5_, exploring the CVD burden attributed to PM_2.5_ is crucial for health resource allocation, policy-making on prevention strategies of chronic diseases and assessment of PM_2.5_ reducing policies. Although several published studies reported the burden of CVD and its related mortality attributed to PM_2.5_, [12,13] they are either of limited scope (focusing only on a single subcategory of CVD or on a small local scale), and none of them considered the burden of CVD-associated disability. Therefore, it is necessary to estimate the burden of CVD (especially the burden of CVD-associated disability) attributed to PM_2.5_ based on a nationally representative database.

By using large national representative data, this study investigated the relationship between long-term exposure to PM_2.5_ and cardiovascular disease-induced disability among Chinese middle-aged and older adults. Furthermore, this research estimated the burden of CVD-associated disability attributed to PM_2.5_. This study can fill the knowledge gap regarding the nonfatal impacts of PM_2.5_-related CVD.

## Methods

### Study population

Participants were from the Second National Sample Survey on Disability (SNSSD), which was implemented by the China State Council from 1 April to 31 May 2006. The SNSSD included retrospective information on the onset time of disabilities and the prevalence, causes, severity of disabilities, living conditions and health service needs. A multistage, stratified random-cluster sampling strategy was used to select 2,526,145 non-institutionalized individuals from 31 provincial-level administrative divisions in mainland China, which are nationally representative of the Chinese population. Within each provincial-level administrative division, sampling strata were defined according to subordinate administrative areas, local geographical characteristics, and local gross domestic product, where appropriate, to allow for anticipated regional variability [14]. Within each stratum, a four-stage sampling strategy was used in this survey, and sampling was conducted with probability proportional to cluster size [14]. The first sampling stage was to select counties from 31 provincial-level administrative divisions in China. The investigators randomly selected counties from each stratum for the next stage of sampling. The following stages were used to randomly select towns from counties, villages/districts from towns, and communities from villages/districts. This survey comprised a total of 734 counties, 2,980 towns, and 5,964 communities. All households in each selected community were investigated. Ultimately, 771,797 households and 2,526,145 individuals were selected for this survey, with a participation rate of 99.8%.

More than 20,000 interviewers, 50,000 survey assistants, and 6,000 doctors of various specialties completed this survey. Trained interviewers accompanied by assistants who are familiar with the communities visited the family members aged seven years or above of each household in the selected community to collect data on demographics and to inquire about visual, hearing, speech, and physical disability by using structured questionnaires. If the interviewees had cognitive impairment or did not have the ability to answer the questionnaire, the questions relevant to them were answered by one of their family members. After that, the designated specialties performed further disability screening and confirmation for those who responded ‘yes’ to any of the corresponding questions and all children aged six years or less [14]. More details of the SNSSD design and sample processing can be found elsewhere [14].

**Figure 1:**
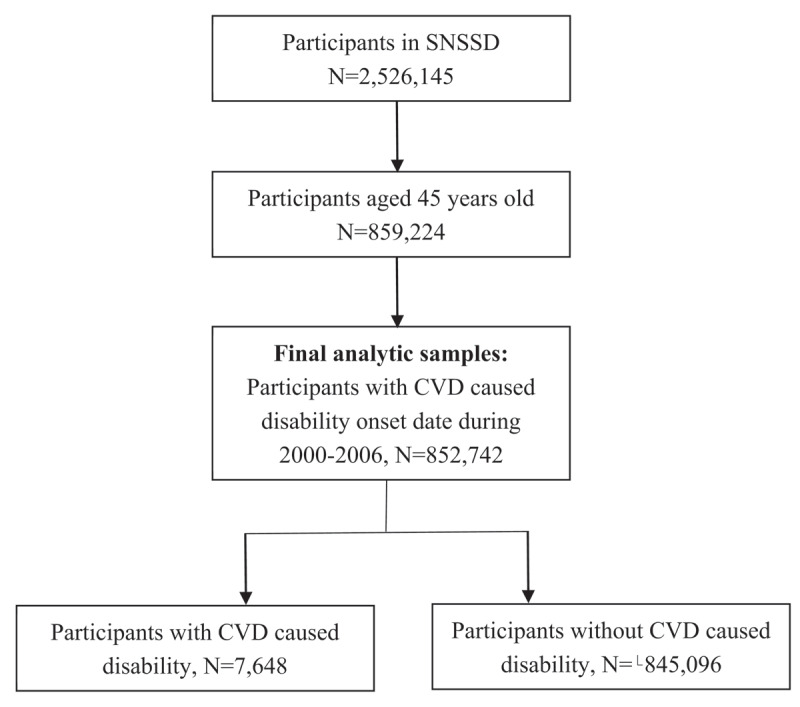
Flow chart of the study sample.

Participants with the information of disability onset date during 2001–2006 were selected in the analysis of this study to maintain the highest level of data accuracy and reduce recall bias. Because the middle-aged and older group is susceptible to CVD by PM_2.5_ [9], this study restricted the analysis to adults aged 45 years or older and finally included 852,742 participants in this study. ***Figure 1*** presents a flow chart of this study.

### Ethics approval

The survey was conducted in all provinces by the Leading Group of the National Sample Survey on Disability and the National Bureau of Statistics. It was approved by the China State Council (No. 20051104) and was implemented within the legal framework governed by the Statistical Law of the People’s Republic of China. All survey respondents provided consent to the Chinese government.

### Measurements

#### Cardiovascular disease-associated disability (CVD)

CVD-associated disability was defined as disability caused by disorders related to heart and blood vessels, which was ascertained by the combination of self-reports or family members’ reports and on-site medical diagnosis by experienced specialists. In the SNSSD, cerebral infarction, cerebral hemorrhage, cerebrovascular disease and peripheral vascular disease were CVD-related causes of disability, was ascertained by the combination of self-reports or family members’ reports and on-site medical diagnosis by experienced specialists. First, trained field interviewers screened persons with disability by using a structured questionnaire with five items during the household face-to-face interview process. This questionnaire was developed for the survey according to the ‘Guidelines and Principles for the Development of Disability Statistics,’ which has been demonstrated to have high reliability [15]. Persons who answered a positive response were labeled as likely to be the suspected cases with disability. Second, specialists with more than five years of clinical experience identified the disability by using The World Health Organization Disability Assessment Schedule, Version II (WHO DAS II) in the suspected cases [16]. Persons who received a score of 52 or higher were diagnosed with disability. Third, experienced specialists diagnosed persons who had any of the following diseases as CVD-associated disability: cerebral infarction, cerebral hemorrhage, cerebrovascular disease or peripheral vascular disease. More details about the criteria and process of diagnosis can be found in previous studies [17,18].

*PM_2.5_ Exposure* Annual average PM_2.5_ concentrations from 2000 to 2006 were used to evaluate the air quality. The dataset of PM_2.5_ was obtained from the spatiotemporal coverage over annual gridded maps of PM_2.5_ in China, which had a spatial resolution of 10 km × 10 km. These maps were estimated by using the machine learning method based on the observations of PM_2.5_ from 1497 sites covering mainland China from 2013–2016, the satellite measurements of aerosol optical depth from 2000–2016, and Community Multiscale Air Quality Model simulations from emission inventories [19]. The estimation was found to have high consistency with the in situ observations [19]. More details about the estimation can be found in a previous publication [19].

PM_2.5_ exposure levels were estimated at the county level for the restriction of geographic information. To evaluate the long-term exposure to PM_2.5_ and the onset of CVD-associated disability, the average PM_2.5_ concentrations (based on the average value estimated across the onset year and its previous year) were assigned to residential postal codes throughout the county in the SNSSD database during the disability onset date from 2001 to 2006. The selected clusters throughout China and the corresponding boundaries of administrative divisions can be found in Appendix Figure 1, and maps with the mean air PM_2.5_ concentrations can be found in Appendix Figure 2.

### Measures

The outcome variable was a binary measure (i.e., whether CVD-associated disability was present), and the independent variable was PM_2.5_. Control variables included age (continuous variable), sex (male or female), residence (rural or urban), marital status (unmarried/married), education (primary school and below or above), household income per capita (Tertile 1 [the lowest household income per capita], Tertile 2 or Tertile 3 [the highest household income per capita]), and electricity consumption per person per month (continuous variable).

### Statistical analysis

Logistic regression models were used to examine the relationship between PM_2.5_ and CVD-associated disability, allowing for multiple demographic and socioeconomic covariates and fixed effects of each province. In addition, nonlinear associations between PM_2.5_ and CVD-associated disability were also presented. To evaluate the non-linear concentration-response relationship, this study modelled PM_2.5_ air pollution using restricted cubic splines with knots at the 5^th^, 35^th^, 65^th^, and 95^th^ percentiles of the distribution of PM_2.5_ concentrations. The exposure level was set at the 35^th^ percentile of the distribution of PM_2.5_ concentrations. Additionally, both population attributable risk (PAR) and its related case number were used to describe the burden of CVD-related disability attributed to high PM_2.5_ exposure. A *P* value less than 0.05 was considered statistically significant. The software Stata version 13.0 for Windows (Stata Corp, College Station, TX, USA) was utilized for statistical analysis.

## Results

### Characteristics of participants

***Table 1*** shows the demographic and socioeconomic features of the participants. Compared with samples in areas with long-term PM_2.5_ concentrations < 75 µg/m^3^, samples in areas with long-term PM_2.5_ concentrations ≥ 75 µg/m^3^ had a higher prevalence of CVD-associated disability. Urban dwellers and unmarried individuals are more likely to reside in places with higher PM_2.5_ concentrations. Residents in areas with long-term PM_2.5_ concentrations ≥ 35 µg/m^3^ had higher education levels, household income per capita, and higher consumption of electricity per person per month than residents in areas with long-term PM_2.5_ concentrations < 35 µg/m^3^. More details can be found in ***Table 1***.

**Table 1:** Characteristics of participants.


CHARACTERISTICS	LEVEL OF PM_2.5_, N (N %)		

	0–35 μg/m^3^	35–75 μg/m^3^	≥75 μg/m^3^

CVD caused disability			

No	253592(99.18)	293979(99.29)	297525(98.86)

Yes	2106(0.82)	2108(0.71)	3434(1.14)

Age (mean (IQR), yr)	59.00(16)	59.12(15)	59.09(15)

Sex			

Male	127070(49.70)	147892(49.95)	147890(49.14)

Female	128628(50.30)	148195(50.05)	153069(50.86)

Residence			

Rural	178710(69.89)	187214(63.23)	187440(62.28)

Urban	76988(30.11)	108873(36.77)	113519(37.72)

Education			

Primary school and below	85881(33.59)	79124(26.72)	81810(27.18)

Junior high school and above	169817(66.41)	216963(73.28)	219149(72.82)

Household income per capita			

Tertile 1 (Lowest)	97328(38.06)	86852(29.33)	82013(27.25)

Tertile 2	78537(30.71)	84370(28.50)	86518(28.75)

Tertile 3 (Highest)	79833(31.22)	124865(42.17)	132428(44.00)

Marital status			

Unmarried	207860(81.29)	244009(82.41)	249947(83.05)

Married	47838(18.71)	52078(17.59)	51012(16.95)

Electricity consumption per person per month (mean (IQR), 10kw/h)	1.46(1.35)	1.78(1.75)	1.90(2)


**Figure 2:**
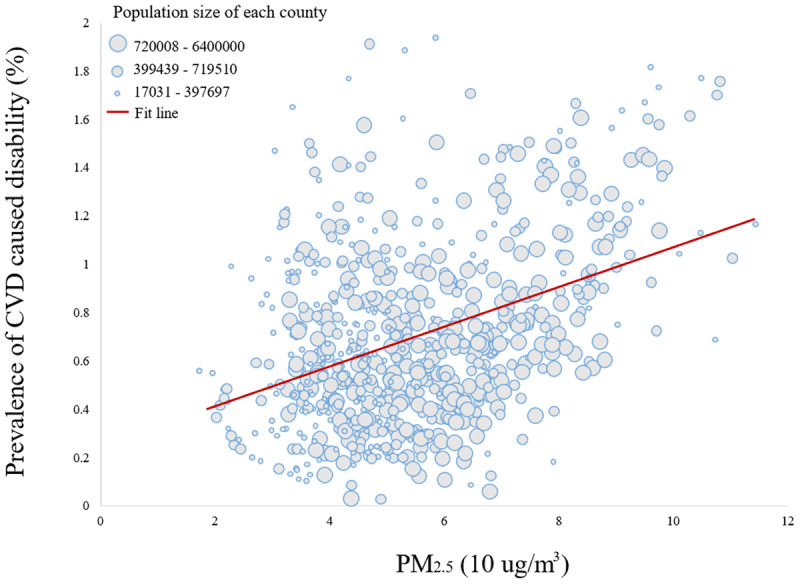
The relationship between the level of exposure to PM_2.5_ concentrations (10 µg/m^3^) and the prevalence of disabilities caused by CVD.

**Figure 3:**
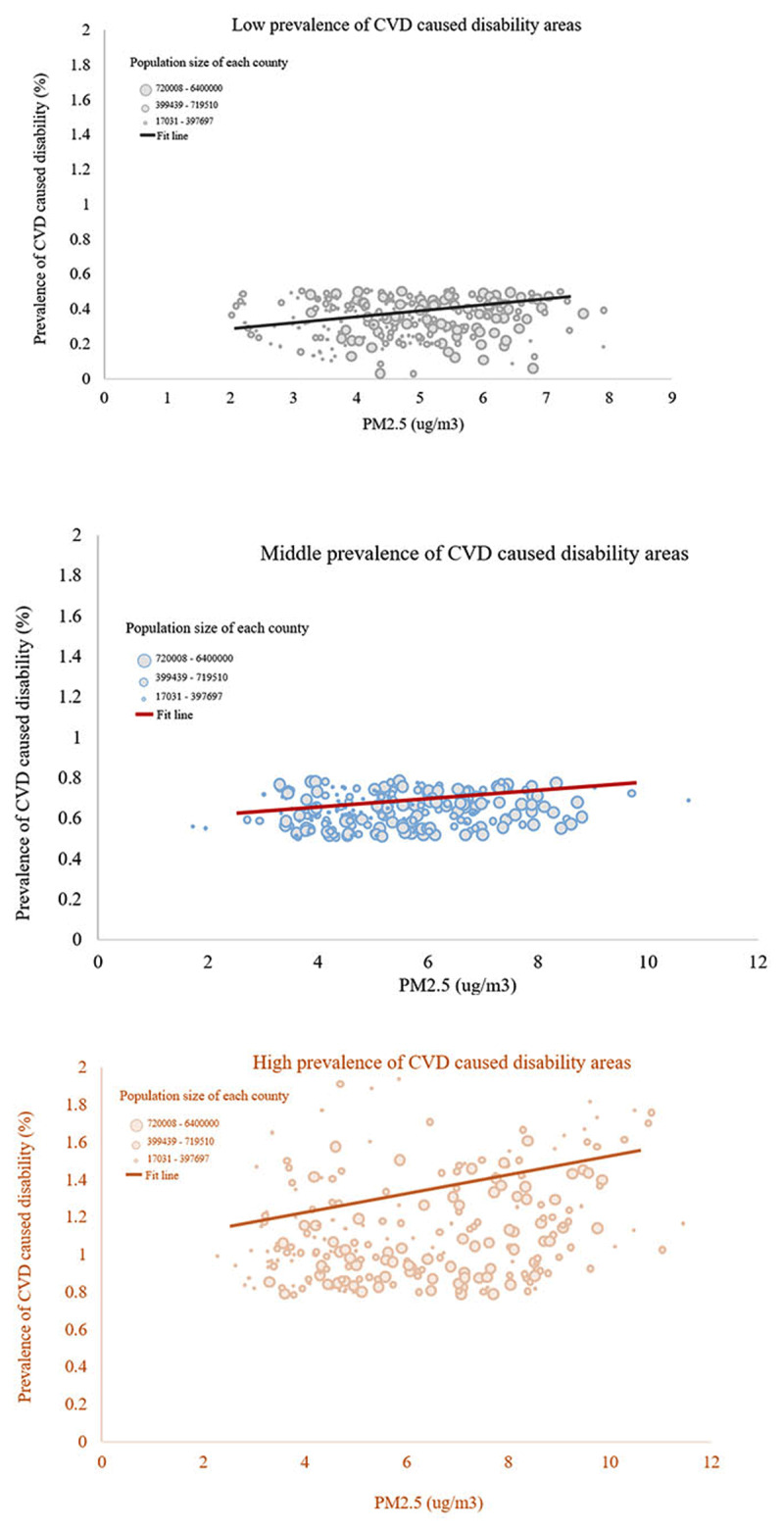
The relationship between level of exposure to PM_2.5_ concentrations (10 ug/m^3^) and prevalence of disabilities caused by CVD, by different areas.

***Figure 2*** presents the relationship between the level of long-term exposure to PM_2.5_ and the prevalence of CVD-associated disability. The fit line showed that a higher prevalence of CVD-associated disability was associated with an increase in PM_2.5_ concentrations. This study also found similar relationships among areas with various prevalences of CVD-associated disability, as shown in ***Figure 3***. A higher magnitude of this relationship was found in places with a higher prevalence of CVD-associated disability.

### The association between long-term PM_2.5_ concentrations and the odds of CVD-associated disability

**Figure 4:**
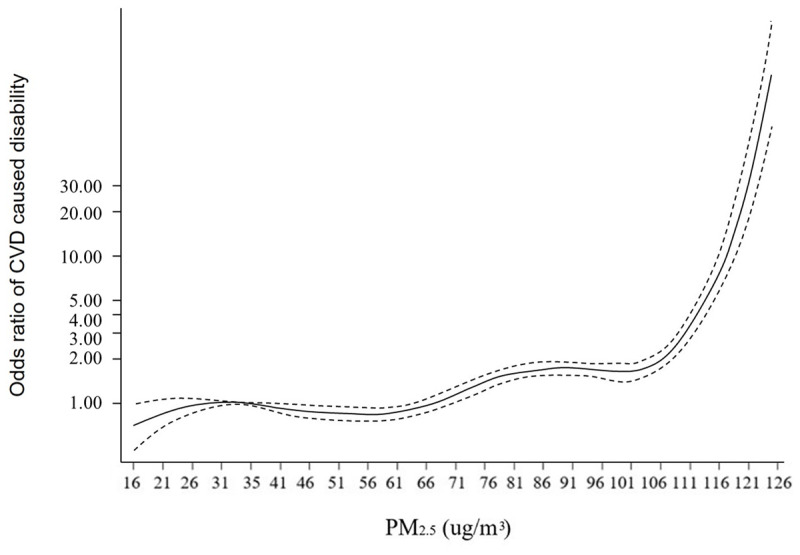
Odds ratios (solid line) with 95% CI (dashed lines) for the relation of PM_2.5_ (µg/m^3^) to the occurrence of disabilities caused by CVD among adults aged 45 years old and above. *Note*: All odds ratios were adjusted for age, gender, residency, marital status, education, household income per capita and electricity consumption per person per month.

The association between CVD-associated disability and PM_2.5_ concentrations is presented in ***Table 2***. The unadjusted odds ratio in the analyses of this study confirmed that incremental changes in PM_2.5_ concentrations tended to be strongly linked to higher odds of CVD-associated disability (OR = 1.13, 95% CI: 1.12, 1.14), which remained robust after controlling for the covariates (OR = 1.27, 95% CI: 1.25, 1.30). Model 3 adjusted for the fixed effect of each province and showed a lower OR (OR = 1.08, 95% CI: 1.05, 1.10) (***Table 2***). Stratified analyses by demographic factors suggested that an increase in PM_2.5_ concentrations was robustly related to a higher likelihood of CVD-associated disability among Chinese middle-aged and older adults (Appendix Table 1). The nonlinear model further confirmed the relationship between higher PM_2.5_ concentrations and the odds of CVD-associated disability, which suggested a relatively steep dose–response function in areas with high PM_2.5_ concentrations (***Figure 4***).

**Table 2:** Estimated odds ratios and 95% CI for disabilities caused by CVD with an increase of 10 µg/m^3^ per PM_2.5_.


CHARACTERISTICS	MODEL 1^a^	MODEL 2^b^	MODEL 3^c^

PM_2.5_ (continuous, 10 µg/m3)	1.13 (1.12, 1.14)	1.27 (1.25, 1.30)	1.08 (1.05, 1.10)

Age (continuous, yr)		1.07 (1.07, 1.07)	1.07 (1.07, 1.08)

Sex			

Male		Reference	Reference

Female		0.75 (0.71, 0.79)	0.73 (0.70, 0.77)

Residence			

Rural		Reference	Reference

Urban		1.38 (1.30, 1.46)	1.30 (1.22, 1.38)

Marital status			

Unmarried		Reference	Reference

Married		0.91 (0.86, 0.96)	0.90 (0.85, 0.96)

Education			

Primary school and below		Reference	Reference

Junior high school and above		0.99 (0.86, 0.96)	0.93 (0.88, 0.99)

Household income per capita			

Tertile 1 (lowest)		Reference	Reference

Tertile 2		0.81 (0.76, 0.85)	0.81 (0.77, 0.86)

Tertile 3 (highest)		0.58 (0.54, 0.62)	0.59 (0.55, 0.64)

Electricity consumption per person per month (continuous, kw/h)		0.99 (0.98, 1.01)	1.00 (0.98, 1.01)

Fixed effects of each province			0.40 (0.31, 0.52)


*Note*: ^a^ Unadjusted model. ^b^ Adjusted for model 2 criteria and age, gender, residency, marital status, education, household income per capita, electricity consumption per person per month, medical insurance. ^c^ Adjusted for model 3 criteria and fixed effect of each province.

### The burdens of CVD-associated disability attributed to high PM_2.5_ concentration exposure

***Table 3*** and Appendix Table 2 show the estimated burdens of CVD-associated disability attributed to ambient particles in Chinese adults aged 45 years old and above. PAR estimation without the fixed effect of each province adjustment showed that 23.92% (22.01%, 25.78%) of CVD-associated disability was attributed to high PM_2.5_ exposure (≥35 µg/m^3^), with 3.53 million cases according to the ambient air quality standards (AAQS) of China [20]. According to WHO guidelines of ambient air quality [21], a high PM_2.5_ concentration (≥10 µg/m^3^) contributed to 45.76% (42.66%, 48.68%) of CVD-related disability cases and 3.53 (3.29,3.75) million cases. The results with the fixed effect of each province adjustment showed that high PM_2.5_ exposure was attributed to 13.59% (9.55, 17.46) and 27.88% (20.12, 34.91) under the Chinese AAQS and WHO guidelines, respectively. The related case numbers were 1.05 (0.74, 1.35) million and 2.15 (1.55, 2.69) million, respectively.

**Table 3:** Estimated burdens of disabilities caused by CVD attributed to ambient particles among Chinese adults aged 45 years old and above.


ATTRIBUTED TO AMBIENT PARTICLES	WITH THE ADJUSTMENT OF FIXED EFFECTS OF EACH PROVINCE

**Chinese guidelines^a^**	

*Total*	

PAR (95% CI), %	13.59 (9.55, 17.46)

Cases attributed to high PM_2.5_ (95% CI), million	1.05(0.74,1.35)

*Low areas* ^b^	

PAR (95% CI), %	8.92(6.22, 11.55)

Cases attributed to high PM^2.5^ (95% CI), million	0.09(0.06,0.12)

*Middle areas* ^c^	

PAR (95% CI), %	12.81 (8.99,16.48)

Cases attributed to high PM_2.5_ (95% CI), million	0.27(0.19,0.34)

*High areas* ^d^	

PAR (95% CI), %	18.81 (13.34, 23.95)

Cases attributed to high PM_2.5_ (95% CI), million	0.69(0.49,0.89)

**WHO guidelines** ^e^	

*Total*	

PAR (95% CI), %	27.88 (20.12, 34.91)

Cases attributed to high PM_2.5_ (95% CI), million	2.15(1.55,2.69)

*Low areas* ^b^	

PAR (95% CI), %	23.98 (17.17, 30.25)

Cases attributed to high PM_2.5_ (95% CI), million	0.26(0.18,0.33)

*Middle areas* ^c^	

PAR (95% CI), %	27.23 (19.62, 34.14)

Cases attributed to high PM_2.5_ (95% CI), million	0.61(0.44,0.77)

*High areas* ^d^	

PAR (95% CI), %	32.23 (23.46, 40.03)

Cases attributed to high PM_2.5_ (95% CI), million	1.28(0.93,1.60)


*Note*: ^a^ Chinese guidelines: annual average PM_2.5_ = 35 µg/m^3^; ^b^ Areas with low prevalence disabilities caused by CVD; ^c^ Areas with middle prevalence disabilities caused by CVD; ^d^ Areas with high prevalence disabilities caused by CVD; ^e^ WHO guidelines: annual average PM_2.5_ = 10 µg/m^3^.

The magnitude of PAR and the number of cases contributing to high PM_2.5_ exposure increased in areas with a high prevalence of CVD-associated disability. For the adjusted results, a reduction of PM_2.5_ to Chinese AAQS corresponded to decreases of 8.92% (6.22%, 11.55%), 12.81% (8.99%, 16.48%), and 18.81% (13.34%, 23.95%) in CVD-associated disability in low, middle, and high prevalence of CVD-related disability, respectively. Reducing PM_2.5_ to WHO guidelines corresponded to 23.98% (17.17%, 30.25%), 27.23% (19.62%, 34.14%) and 32.23% (23.46%, 40.03%) reductions in disabilities in areas with low, middle, and high CVD-related disability prevalence, respectively. More details can be found in ***Table 3***.

## Discussion

This study investigated the relationship between long-term PM_2.5_ exposure and CVD-associated disability among middle-aged and older adults in China and estimated the burden from CVD-associated disability, which provides the opportunity to link air pollution to CVD-related disabilities, which has rarely been done before. To the best of our knowledge, this is the first report about the association of county-level PM_2.5_ concentrations with CVD-associated disability. The results showed that every increase in 10 μg/m^3^ was associated with an 8% increase in the odds of CVD-associated disability after controlling covariates, which is lower than the odds ratios of PM_2.5_ on morbidities of cardiovascular events in previous studies [22,23]. Our findings of the health benefits from compliance with the air quality standard for PM_2.5_ are similar to previous studies in China regarding the role of ambient particles on morbidity and mortality related to CVDs [8,11].

Previous studies have put forward several hypotheses to explain the relationship between cardiovascular events and air pollution. First, PM_2.5_ is associated with biomarkers including systemic inflammation, thrombosis, and endothelial dysfunction, which are associated with elevated fibrinogen, C-reactive protein, white blood cell count, activation markers P-selectin, and soluble CD40 ligand [24,25]. Second, PM_2.5_ is associated with cardiometabolic risk factors, which may accelerate atherosclerosis and increase vulnerability to plaque rupture and correlate with carotid intima-media thickness in humans, which is associated with the risk of heart attack or cerebrovascular disease [11,26,27]. In addition, air pollution may adversely affect measures of cardiovascular physiology, such as heart rate and blood pressure [24]. A steep dose–response function at high PM_2.5_ concentration areas was found in this study, which implied no saturation effect for the odds of CVD-associated disability even at a level of annual PM_2.5_ concentrations as high as 120 μg/m^3^. Sensitivity analysis for the knots placed on the 25th, 40th, 60th and 80th percentiles of the PM_2.5_ concentration and the linear function presented similar results (Appendix Figure 3 and Appendix Figure 4). At lower levels of PM_2.5_, there was no significant relationship between PM_2.5_ concentrations and the increased odds of disabilities of cardiovascular events, but previous evidence showed a steeper dose–response on morbidities of cardiovascular events at lower concentrations [28], adding that there is no threshold for safety of PM_2.5_ pollution and cardiovascular events. Although a shallower response at higher concentrations areas was found in a previous study, for CVD-associated disability in the results of this study, it was a steep dose–response at high levels. These findings imply that PM_2.5_ has a marginal decreasing effect on CVD morbidity at high levels of PM_2.5_, but the cumulative effect of PM_2.5_ on CVD disability increases incrementally. To some extent, it also implies that the severity of CVD may become greater with incremental changes in PM_2.5_, which may increase the probability of the transition from morbidity to disability of CVD at high levels of PM_2.5_.

This study indicated that a high PM_2.5_ concentration could lead to a high burden of CVD-related disability. In total, 1.05 million CVD-related disability cases among Chinese middle-aged and older adults could be attributed to high PM_2.5_ concentration (≥35 µg/m^3^) exposure, which accounted for 34.53% of CVD-related deaths (Estimation of Global Burden of Disease Study in 2006: deaths = 3.08 million) [29]. A reduction in PM_2.5_ concentration to 35 µg/m^3^ corresponded to a decrease of 13.59% in CVD-associated disability in this study, and this magnitude increased in areas with a higher prevalence of CVD-related disability. These results support that reducing the target for PM_2.5_ concentration is significant to promote public health and reduce air pollution-related medical expenditures and rehabilitation fees, and the effectiveness of the PM_2.5_ reducing strategies would be more significant in areas with a high burden of CVD-related disability.

### Limitations

This study has a number of limitations. First, the results should be interpreted with caution because the onset of CVD-related disability was based on self-reported retrospective information, and the investigation only involved part of the CVD-related disorders, such as cerebral infarction, cerebral hemorrhage, cerebrovascular disease and peripheral vascular disease. Second, some variables, such as dietary structure, smoking status and the average time to healthcare facility, were not considered due to data restrictions, which may become confounders of the response relationship between PM_2.5_ and CVD-associated disability. Third, for the data constraints, the categorization of CVD in this study was quite general and did not differentiate the types of CVD. Fourth, this study controls the fixed effect of each province in the analysis, which could control some unmeasured confounders that spatially varied. However, it might also be correlated with the exposure variable, which resulted in potential collinearity and thus underestimation of the association. Fifth, due to the lack of monitoring data on ground surface PM_2.5_ during our study period in China, the exposure was evaluated based on the estimated concentrations from the satellite remote sensing measurements and historical emission inventories. The uncertainty in the estimated concentrations of PM_2.5_ could increase the probability of exposure misclassification, which might increase bias in our findings. Furthermore, some necessary conditions in each air pollution cell, such as population weight and house-moving factors, were not considered in the county-level PM_2.5_ concentration estimation, which could bias our results. Therefore, future studies should perform further investigation to verify the results.

## Conclusions

The research findings suggested that incremental changes in PM_2.5_ were strongly linked to higher odds of CVD-associated disability in China, and there was a steep dose–response function in areas with high PM_2.5_ concentrations by using a vast dataset covering China. Among Chinese middle-aged and older adults in 2006, 1.05–3.53 million (13.59–23.92%) CVD-associated disabilities were attributed to high PM_2.5_ concentrations (≥35 µg/m^3^). The findings in this study suggested that reducing PM_2.5_ concentrations may contribute to CVD-associated disability prevention. Clinicians should be aware not only of the increased odds of elevated CVD at high levels of PM_2.5_ but also of the increased probability of the transition from morbidity to disability caused by CVD. In the future, more studies should focus on the linkage of air pollution to CVD disability. Continued improvements in air pollution abatement are important not only for decreasing the odds of CVD- or CVD-caused mortality but also for CVD disability prevention.

## Additional Files

The additional files for this article can be found as follows:

10.5334/gh.1118.s1 Table 1 Estimated odds ratios and 95% CI for disabilities caused CVD with an increase of 10 µg/m^3^ PM_2.5_, by demographic characteristics. Stratified analyses by demographic factors suggested that an increase in PM_2.5_ concentrations was robustly related to a higher likelihood of CVD-associated disability among Chinese middle-aged and older adults.

10.5334/gh.1118.s2 Table 2 Estimated burdens of disabilities caused by CVD attributed to ambient particles among Chinese adults aged 45 years old and above. The estimated burdens of CVD-associated disability attributed to ambient particles in Chinese adults aged 45 years old and above.

10.5334/gh.1118.s3 Figure 1 Map with the selected clusters throughout China. The selected clusters throughout China and the corresponding boundaries of administrative divisions could be found.

10.5334/gh.1118.s4 Figure 2 Maps of annual average concentrations of PM_2.5_ in China, 2000–2006]. Maps with the mean air PM_2.5_ concentrations.
