# Supplementary material for: PM2.5 Air Pollution and Cardiovascular Disease-Associated Disability among Middle-Aged and Older Adults
Source: Glob Heart. 2022 Jun 16;17(1):41. doi: 10.5334/gh.1118 (PMC9205374; doi:10.5334/gh.1118)
Supplement: Table 1 Estimated odds ratios and 95% CI for disabilities caused CVD with an increase of 10 µg/m3 PM2.5, by demographic characteristics. — Stratified analyses by demographic factors suggested that an increase in PM2.5 concentrations was robustly related to a higher likelihood of CVD-associated disability among Chinese middle-aged and older adults. [file gh-17-1-1118-s1.pdf]

Appendix

Table 1 Estimated odds ratios and 95% CI for disabilities caused CVD with an increase of 10 μg/m^3 $PM_{2.5}$, by demographic characteristics

**Characteristics**	$PM_{2.5}$ (continuous, 10 μg/m3)
Gender	
Female	1.10(1.06,1.14)
Male	1.06(1.03,1.10)
Residence	
Urban	1.07(1.03,1.11)
Rural	1.09(1.06,1.12)
Age group	
45-64	1.10(1.07,1.14)
65-84	1.06(1.03,1.10)
85+	1.12(1.03,1.21)

Note: All odds ratios adjusted for age, gender, residency, marital status, education, household income per capita, electricity consumption per person per month and the spatial random effect.
