# Supplementary material for: PM2.5 Air Pollution and Cardiovascular Disease-Associated Disability among Middle-Aged and Older Adults
Source: Glob Heart. 2022 Jun 16;17(1):41. doi: 10.5334/gh.1118 (PMC9205374; doi:10.5334/gh.1118)
Supplement: Table 2 Estimated burdens of disabilities caused by CVD attributed to ambient particles among Chinese adults aged 45 years old and above. — The estimated burdens of CVD-associated disability attributed to ambient particles in Chinese adults aged 45 years old and above. [file gh-17-1-1118-s2.pdf]

Table 2 Estimated burdens of disabilities caused by CVD attributed to ambient particles among Chinese adults aged 45 years old and above

Attributed to ambient particles	Without the adjustment of fixed effect of each province
**Chinese guidelines** [a]	
Total	
PAR(95%CI),%	23.92 (22.01, 25.78)
Cases attributed to high $PM_{2.5}$ (95%CI), million	1.84(1.70,1.99)
Low areas [b]	
PAR(95%CI),%	16.04 (14.70, 17.36)
Cases attributed to high $PM_{2.5}$ (95%CI), million	0.16(0.15,0.18)
Middle areas [c]	
PAR(95%CI),%	22.63(20.81,24.41)
Cases attributed to high $PM_{2.5}$ (95%CI), million	0.48(0.44,0.52)
High areas [d]	
PAR(95%CI),%	32.29(29.86,34.65)
Cases attributed to high $PM_{2.5}$ (95%CI), million	1.21(1.11,1.30)
**WHO guidelines** [e]	
Total	
PAR(95%CI),%	45.76 (42.66, 48.68)
Cases attributed to high $PM_{2.5}$ (95%CI), million	3.53(3.29,3.75)
Low areas [b]	
PAR(95%CI),%	40.14(37.29,42.86)
Cases attributed to high $PM_{2.5}$ (95%CI), million	0.44(0.41,0.47)
Middle areas [c]	
PAR(95%CI),%	44.84(41.78,47,74)
Cases attributed to high $PM_{2.5}$ (95%CI), million	1.01(0.94,1.08)
High areas [d]	
PAR(95%CI),%	51.73(48.43,54.81)
Cases attributed to high $PM_{2.5}$ (95%CI), million	2.08(1.94,2.20)

Note:

[a] Chinese guidelines: annual average $PM_{2.5}$=35μg/m^3;

[b] Areas with low prevalence disabilities caused by CVD;

[c] Areas with middle prevalence disabilities caused by CVD;

[d] Areas with high prevalence disabilities caused by CVD;

[e] WHO guidelines: annual average $PM_{2.5}$=10 μg/m^3.
