# Supplementary figures and images for: PM2.5 Air Pollution and Cardiovascular Disease-Associated Disability among Middle-Aged and Older Adults
Source: Glob Heart. 2022 Jun 16;17(1):41. doi: 10.5334/gh.1118 (PMC9205374; doi:10.5334/gh.1118)

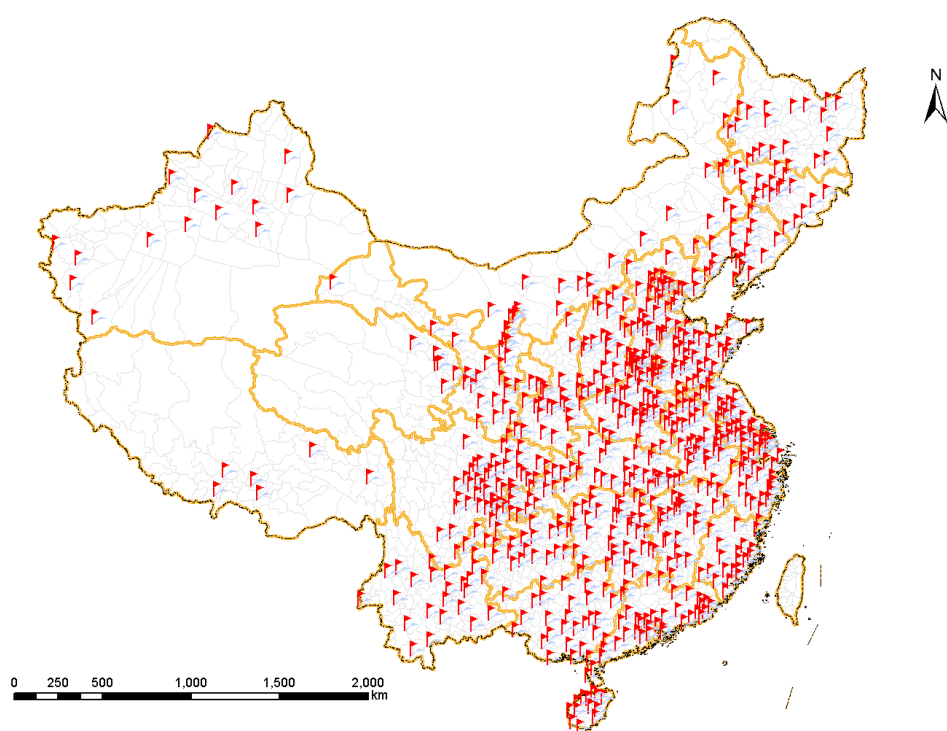

**Figure 1** Map with the selected clusters throughout China

Supplement: Figure 1 Map with the selected clusters throughout China. — The selected clusters throughout China and the corresponding boundaries of administrative divisions could be found. [file gh-17-1-1118-s3.pdf]

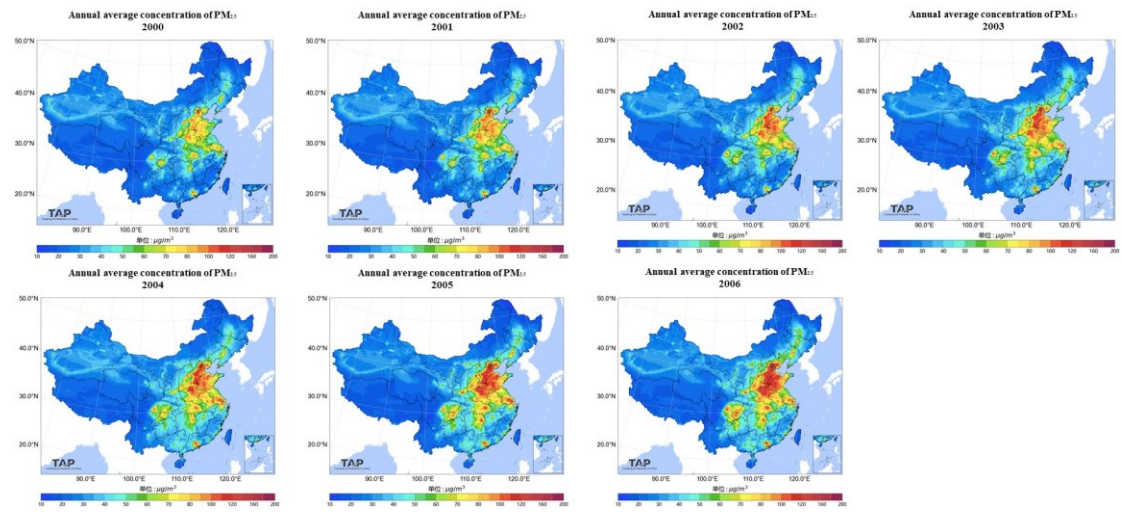

**Figure 2** Maps of annual average concentrations of PM<sub>2.5</sub> in China, 2000-2006

Supplement: Figure 2 Maps of annual average concentrations of PM2.5 in China, 2000–2006]. — Maps with the mean air PM2.5 concentrations. [file gh-17-1-1118-s4.pdf]
